# Association between serum netrin-1 levels and early neurological deterioration after acute ischemic stroke

**DOI:** 10.3389/fneur.2022.953557

**Published:** 2022-08-24

**Authors:** Zhuo Chen, Tianli Cao, Xingju Zhong, Yong Wu, Wei Fu, Chaoli Fan, Yu Jiang, Qi Zhou, Jie Peng, Jieyu Liao, Zhike You, Xin Yi, Jingyu Tan

**Affiliations:** ^1^Department of Neurology, Mianzhu People's Hospital, Mianzhu, China; ^2^Department of Endocrinology, Mianzhu People's Hospital, Mianzhu, China

**Keywords:** acute ischemic stroke, early neurological deterioration, biomarker, netrin-1, restricted cubic spline

## Abstract

**Background and purposes:**

Experimental studies demonstrated that netrin-1 (NT-1) has anti-inflammatory, tissue regeneration, and immune modulation properties. We aimed to discern the utility of NT-1 as a biomarker for assessing the risk of early neurological deterioration (END) after ischemic stroke.

**Methods:**

This was a prospective study enrolling ischemic stroke patients with symptoms onset <24 h. Serum NT-1 concentrations were measured at admission. The National Institutes of Health Stroke Scale increased by ≥2 points and ≥4 points during the first 72 h after admission and was defined as END2 and END4, respectively.

**Results:**

The study included 268 patients (146 men and 122 women) with a mean age of 63.0 ± 9.6 years. The median NT-1 concentrations were 466.4 pg/ml (interquartile range, 341.4–589.2 pg/ml). During the initial 72 h after admission, END2 was found in 83 (31.0%) patients, and END4 was observed in 48 (17.9%) subjects. After adjusted for potential confounders, multivariate analysis indicated that decreased NT-1 levels is an independent predictor for END2 [odds ratio (*OR*) 0.62, 95% confidence interval (*CI*) 0.46–0.84, *p* < 0.001) and END4 (*OR* 0.53, 95% *CI* 0.36–0.76, *p* < 0.001). Similar results were found when the NT-1 levels were analyzed as a categorical variable. Furthermore, restricted cubic spline analysis showed a linear association between NT-1 concentrations and the risk of END (END2, *p* = 0.006 for linearity; END4, *p* < 0.001 for linearity).

**Conclusions:**

Our results suggest that decreased NT-1 levels were significantly associated with a higher risk of END after ischemic stroke.

## Introduction

Ischemic stroke is a leading cause of death and adult disability globally, exerting a heavy economic burden (1). According to the literature, approximately one-third of patients with acute ischemic stroke (AIS) would experience early neurological deterioration (END) during hospitalization despite standard treatment (2–4). END is frequently associated with an increased risk of functional disability and mortality (5–7). Therefore, to effectively improve the ischemic stroke outcome, it is essential to accurately identify the risk factor of END after AIS.

Netrin-1 (NT-1) is a bifunctional molecule, identified as a neuronal guidance cue, and directs axons to its targets during the development of the nervous system (8, 9). NT-1 receptors include the deleted in colorectal cancer and uncoordinated-5 families, which belonged to the transmembrane immunoglobulin superfamily (10). It has been reported that NT-1 is involved in several physiological and pathological processes such as dysfunction of the blood–brain barrier, apoptosis, neuroinflammation, and neurogenesis in animal models of cerebral ischemia (11–13). In addition, the overexpression of NT-1 by adeno-associated viral could increase peri-infarct blood vessel density and improve motor function recovery after experimental stroke (14), indicating that NT-1 might play an important role in mediating cerebral injury after ischemic stroke. Recently, several clinical studies have evaluated the prognostic value of NT-1 in ischemic stroke. According to the data from the China Antihypertensive Trial in AIS, decreased baseline NT-1 was significantly correlated to a higher risk of the 90-day composite outcome of death or major disability, and there was a negative linear dose–response relationship between them (15). We also confirmed the important role of NT-1 in predicting depression at 3 months after ischemic stroke (16). However, to date, the association between NT-1 levels and AIS complications, such as END, has not been well-clarified. Therefore, we aimed to prospectively assess the relationship between NT-1 levels and the risk of END in patients with AIS.

## Materials and methods

### Study population

From July to December 2021, consecutive patients who were admitted to the Mianzhu People's Hospital due to first-ever AIS within 24 h of symptom onset were prospectively recruited. AIS was diagnosed according to the World Health Organization criteria (17) and was sequentially confirmed by brain computed tomography or magnetic resonance confirmation. We then excluded participants based on the following exclusion criteria: (1) age <18 years; (2) received the intravenous thrombolysis and/or endovascular treatment after admission; (3) discharged early within 3 days after admission. This study was approved by the Ethics Review Board of Mianzhu People's Hospital. Written informed consent was obtained from each participant or proxy respondent.

### Clinical data collection

We acquired clinical data from each patient, such as demographic characteristics, body mass index (BMI), vascular risk factors, medication history, stroke severity, stroke etiology, and initial hematological findings such as uric acid, hypersensitive C-reactive protein (Hs-CRP), and baseline glucose levels. The severity of the index stroke was evaluated by the National Institutes of the Health Stroke Scale (NIHSS) (18). Ischemic stroke was further classified with reference to TOAST (Trial of Org 10172 in Acute Stroke Treatment) criteria (19).

### Assessment of END

The stroke severity was evaluated by an experienced neurologist using NIHSS at admission and continued 1–3 times a day for 72 h. In our study, we used two previously recommended END definitions to diagnose the presence of END. The first definition (END2) is that the total NIHSS score increased at least two points compared with the baseline NIHSS score (2, 20). The second definition (END4) is that the total NIHSS score increased at least four points compared with the baseline NIHSS score (20, 21).

### Determination of NT-1 levels

Blood was sampled from each subject within 24 h after admission. The specimens were centrifuged at 1,500 g for 10 min and the isolated serum was frozen at −80°C for later analysis. NT-1 levels were measured using commercially available enzyme-linked immunosorbent assay kits according to manufacturers' instructions, by laboratory technicians who were blinded to clinical data.

### Statistical analyses

Categorical variables are expressed as the number of patients (%) and were compared using the chi-square test. Continuous variables are expressed as mean (standard deviation, *SD*) or median (interquartile range, *IQR*) for the continuous variables. Student's *t*-test, the Mann–Whitney *U* test, the one-way analysis of variance, and the Kruskal–Wallis *H* test were applied to compare continuous variables between groups as appropriate. A binary logistic regression analysis was conducted to evaluate the association between NT-1 concentrations and the risk of END. A further multivariate model was computed after adjusting for demographic characteristics and variables with *p* < 0.1 in the univariate analysis. The results are demonstrated as odds ratio (*OR*) with 95% confidence intervals (*CI*). We also used the multiple imputations with chain equations to account for missing values.

We further explored the pattern and magnitude of the association of NT-1 levels with END using restricted cubic splines with 3 knots (at 5th, 50th, and 95th percentiles) adjusted for potential covariates (22). All statistical analysis was conducted with SPSS for Windows version 23.0 (SPSS Inc., Chicago, IL, USA) and R statistical software version 4.0.0 (R Foundation, Vienna, Austria). A two-sided *p*-value of < 0.05 was considered to be statistically significant.

## Results

We evaluated a total of 268 patients with AIS (mean age: 63.0 years; male: 54.5%; median NIHSS score: 6.0). In the study population, 190 patients had hypertension, 77 had diabetes mellitus, 43 had hyperlipidemia, and 22 patients had coronary heart disease. The median levels of NT-1 in this cohort were 466.4 pg/ml, with quartile levels as follows: first quartile (<342.8 pg/ml), second quartile (342.8–466.3 pg/ml), third quartile (466.4–589.0 pg/ml), and fourth quartile (>589.0 pg/ml). The demographic characteristics and clinical and laboratory data are summarized in Table 1. Patients with lower NT-1 levels showed significantly older age (*p* = 0.023), higher prevalence of diabetes (*p* = 0.002), END2 (*p* = 0.009) and END4 (*p* = 0.008), higher levels of baseline glucose (*p* = 0.046) and Hs-CRP (*p* = 0.002), as compared with those with higher serum NT-1 levels.

**Table 1:** Baseline data according to the quartile of netrin-1 levels.

**Variables**	**First quartile** ***n*** **= 67**	**Second quartile** ***n*** **= 67**	**Third quartile** ***n*** **= 67**	**Fourth quartile** ***n*** **= 67**	***P*** **-value**
**Demographic characteristics**					
Age, year	66.1 ± 8.8	62.0 ± 9.7	61.5 ± 8.9	62.5 ± 10.6	0.023
Male, *n* (%)	38 (56.7)	35 (52.2)	38 (56.7)	35 (52.2)	0.910
BMI, kg/m^2^	24.6 ± 2.7	24.3 ± 2.3	25.0 ± 2.8	24.5 ± 2.9	0.510
**Vascular risk factors**, ***n*** **(%)**					
Hypertension	48 (71.6)	46 (68.7)	46 (68.7)	50 (74.6)	0.850
Diabetes	30 (44.8)	18 (26.9)	19 (28.4)	10 (14.9)	0.002
Hyperlipidemia	7 (10.4)	10 (14.9)	14 (20.9)	12 (17.9)	0.397
Coronary heart disease	8 (11.9)	4 (6.0)	6 (9.0)	5 (7.5)	0.645
Current smoking	29 (43.3)	29 (43.3)	26 (38.8)	21 (31.3)	0.444
Current alcohol intake	18 (26.9)	18 (26.9)	19 (28.4)	14 (20.9)	0.765
**Clinical parameters**					
Systolic blood pressure, mmHg	136.9 ± 16.2	138.4 ± 17.9	137.9 ± 15.6	132.9 ± 14.7	0.189
Diastolic blood pressure, mmHg	82.0 ± 10.7	80.7 ± 10.3	81.7 ± 9.3	79.0 ± 8.5	0.287
NIHSS score at admission, score	8.0 (5.0, 10.0)	7.0 (4.0, 10.0)	6.0 (4.0, 9.0)	6.0 (4.0, 9.0)	0.058
Previous statin therapy, *n* (%)	12 (17.9)	17 (25.4)	23 (34.3)	19 (28.4)	0.186
Previous antiplatelet therapy, *n* (%)	24 (35.8)	21 (31.3)	25 (37.3)	19 (28.4)	0.675
END2, *n* (%)	29 (43.3)	22 (32.8)	21 (31.3)	11 (16.4)	0.009
END4, *n* (%)	21 (31.3)	11 (16.4)	9 (13.4)	7 (10.4)	0.008
**Lesion location**, ***n*** **(%)**					0.196
Frontal	8 (11.9)	8 (11.9)	13 (19.4)	20 (29.9)	
Parietal	15 (22.4)	12 (17.9)	7 (10.4)	7 (10.4)	
Basal ganglia	20 (29.9)	19 (28.4)	23 (34.3)	21 (31.3)	
Infratentorial	12 (17.9)	10 (14.9)	10 (14.9)	7 (10.4)	
Other	12 (17.9)	18 (26.9)	14 (20.9)	12 (17.9)	
**Stroke subtypes**, ***n*** **(%)**					0.208
Atherosclerosis	30 (44.8)	33 (49.4)	26 (38.8)	21 (31.3)	
Cardioembolism	9 (13.4)	14 (20.9)	17 (25.4)	16 (23.9)	
Small vessel occlusion	22 (32.8)	17 (25.4)	15 (22.4)	25 (37.3)	
Other	6 (9.0)	3 (4.5)	9 (13.4)	5 (7.5)	
**Laboratory data**					
Baseline glucose levels, mmol/L	6.1 ± 2.7	5.2 ± 1.9	6.1 ± 1.9	5.4 ± 2.3	0.046
Hs-CRP, mg/L	7.5 (2.2, 12.0)	3.5 (1.9, 8.6)	5.8 (2.7, 9.9)	3.3 (1.6, 5.5)	0.002
Uric acid, mmol/L	323.7 ± 97.3	309.2 ± 99.9	315.8 ± 107.7	303.4 ± 82.5	0.662

Hs-CRP, Hyper-sensitive C-reactive protein; NIHSS, National Institutes of Health Stroke Scale; PSD, post-stroke depression.

During the hospitalization, a total of 83 (31.0%) patients experienced END2, and 48 (17.9%) subjects experienced END4. The clinical variables according to patients with and without END are shown in Table 2. In general, patients with END2 and END4 had a higher prevalence of diabetes, more severe baseline neurological deficits, higher levels of baseline glucose levels, and lower NT-1 levels. Furthermore, patients with END 2 were older than patients without (*p* = 0.048). Hypertension was more prevalent in patients with END2 than in patients without (*p* = 0.008). The baseline NIHSS score (*p* = 0.048) and the Hs-CRP levels (*p* = 0.009) were higher in patients with END2 than in patients without END2. After adjustment for age, sex, baseline NIHSS score, and other potential confounders, multivariate analysis showed that patients with the lowest quartile of NT-1 levels were significantly associated with a higher risk of END2 (as compared with higher quartile of NT-1 levels; *OR*, 3.46; 95% *CI*, 1.44–8.31; *p* = 0.005) and END4 (as compared with higher quartile of NT-1 levels; *OR*, 3.18; 95% *CI*, 1.16–8.74; *p* = 0.025). Similar results were found when the NT-1 levels were analyzed as a continuous variable (Table 3).

**Table 2:** Univariate analysis of the factors with early neurological deterioration.

**Variables**	**END2**		***P*** **-value**	**END4**		***P*** **-value**
	**Yes, *n* = 83**	**No, *n* = 185**		**Yes, *n* = 48**	**No, n = 220**	
**Demographic characteristics**						
Age, year	64.8 ± 8.3	62.2 ± 10.1	0.048	64.1 ± 8.3	62.8 ± 9.9	0.387
Male, *n* (%)	45 (54.2)	101 (54.6)	0.954	25 (52.1)	121 (55.0)	0.713
BMI, kg/m^2^	24.3 ± 2.6	24.7 ± 2.7	0.252	24.2 ± 2.6	24.7 ± 2.7	0.298
**Vascular risk factors**, ***n*** **(%)**						
Hypertension	68 (81.9)	122 (65.9)	0.008	37 (77.1)	153 (69.5)	0.288
Diabetes	32 (38.6)	45 (24.3)	0.017	20 (41.7)	57 (25.9)	0.029
Hyperlipidemia	14 (16.9)	29 (15.7)	0.806	7 (14.6)	36 (16.4)	0.761
Coronary heart disease	7 (8.4)	16 (8.6)	0.954	4 (8.3)	19 (8.6)	0.946
Current smoking	37 (44.6)	68 (36.8)	0.225	18 (37.5)	87 (39.5)	0.793
Current alcohol intake	26 (31.3)	43 (23.2)	0.162	11 (22.9)	58 (26.4)	0.621
**Clinical parameters**						
Systolic blood pressure, mmHg	138.1 ± 16.6	135.8 ± 16.0	0.244	138.5 ± 17.0	136.1 ± 16.0	0.346
Diastolic blood pressure, mmHg	81.6 ± 9.2	80.5 ± 10.0	0.411	81.2 ± 8.2	80.8 ± 10.1	0.829
NIHSS score at admission, score	7.0 (5.0, 10.0)	6.0 (4.0, 9.0)	0.048	7.5 (5.0, 12.0)	6.0 (4.0, 9.0)	0.003
Previous statin therapy, *n* (%)	24 (28.9)	47 (25.4)	0.547	12 (25.0)	59 (26.8)	0.796
Previous antiplatelet therapy, *n* (%)	31 (37.3)	58 (31.4)	0.335	16 (33.3)	73 (33.2)	0.984
Lesion location, *n* (%)			0.960			0.977
Frontal	14 (16.9)	35 (18.9)		8 (16.7)	41 (18.6)	
Parietal	13 (15.7)	28 (15.1)		8 (16.7)	33 (15.0)	
Basal ganglia	25 (30.1)	58 (31.4)		15 (31.3)	68 (30.9)	
Infratentorial	14 (16.9)	25 (13.5)		8 (16.7)	31 (14.1)	
Other	17 (20.5)	39 (21.1)		9 (18.8)	47 (21.4)	
Stroke subtypes, *n* (%)			0.414			0.932
Atherosclerosis	36 (43.4)	24 (40.0)		20 (41.7)	90 (40.9)	
Cardioembolism	20 (24.1)	36 (19.5)		10 (20.8)	46 (20.9)	
Small vessel occlusion	23 (27.7)	56 (30.3)		15 (31.3)	64 (29.1)	
Other	4 (4.8)	19 (10.3)		3 (6.3)	20 (9.1)	
**Laboratory data**						
Baseline glucose levels, mmol/L	6.1 ± 2.6	5.5 ± 2.0	0.033	6.6 ± 3.0	5.5 ± 2.0	<0.001
Hs-CRP, mg/L	6.5 (2.6, 9.9)	3.6 (1.9, 8.0)	0.009	6.9 (2.1, 10.0)	3.8 (2.1, 8.5)	0.061
Uric acid, mmol/L	317.4 ± 102.7	311.0 ± 94.6	0.621	302.7 ± 117.8	315.1 ± 92.2	0.431
Netrin-1 level, pg/mL	432.5 (290.3, 490.2)	476.9 (392.8, 600.0)	<0.001	389.8 (276.2, 489.1)	474.4 (391.2, 590.4)	<0.001
Netrin-1 quartiles, *n* (%)			0.009			0.008
First	29 (34.9)	38 (20.5)		21 (43.8)	46 (20.9)	
Second	22 (25.8)	45 (24.3)		11 (22.9)	56 (25.5)	
Third	21 (25.3)	46 (24.9)		9 (18.8)	58 (26.4)	
Fourth	11 (13.3)	56 (30.3)		7 (14.6)	60 (27.3)	

Hs-CRP, Hyper-sensitive C-reactive protein; NIHSS, National Institutes of Health Stroke Scale; PSD, post-stroke depression.

**Table 3:** Multivariate analysis of the association between netrin-1 levels and early neurological deterioration.

**Variables**	**Univariate logistic regression analysis**		**Multivariate logistic regression analysis**	
	**OR (95%CI) for END2**	**OR (95%CI) for END4**	**OR (95%CI) for END2**	**OR (95%CI) for END4**
Each SD increase in Log NT-1	0.59 (0.45–0.78)^§^	0.51 (0.36–0.71)^§^	0.62 (0.46–0.84)^§^	0.53 (0.36–0.76)^§^
**Netrin-1 quartiles**				
First	3.88 (1.73–8.71)^§^	3.91 (1.53–9.99)^§^	3.46 (1.44–8.31)^§^	3.18 (1.16–8.74)^#^
Second	2.49 (1.09–5.67)^#^	3.91 (1.53–9.99)	2.63 (1.10–6.29)^#^	1.59 (0.54–4.67)
Third	2.32 (1.02–5.32)^#^	1.33 (0.47–3.18)	2.40 (1.01–5.74)^#^	1.14 (0.38–3.40)
Fourth	Reference	Reference	Reference	Reference

Multivariate logistic regression analysis was controlled for demographic characteristics, hypertension, baseline NIHSS score, baseline glucose levels and hyper-sensitive C-reactive protein levels.

# Indicates P-value < 0.05;

§ indicates P-value < 0.01.

**Figure 1:**
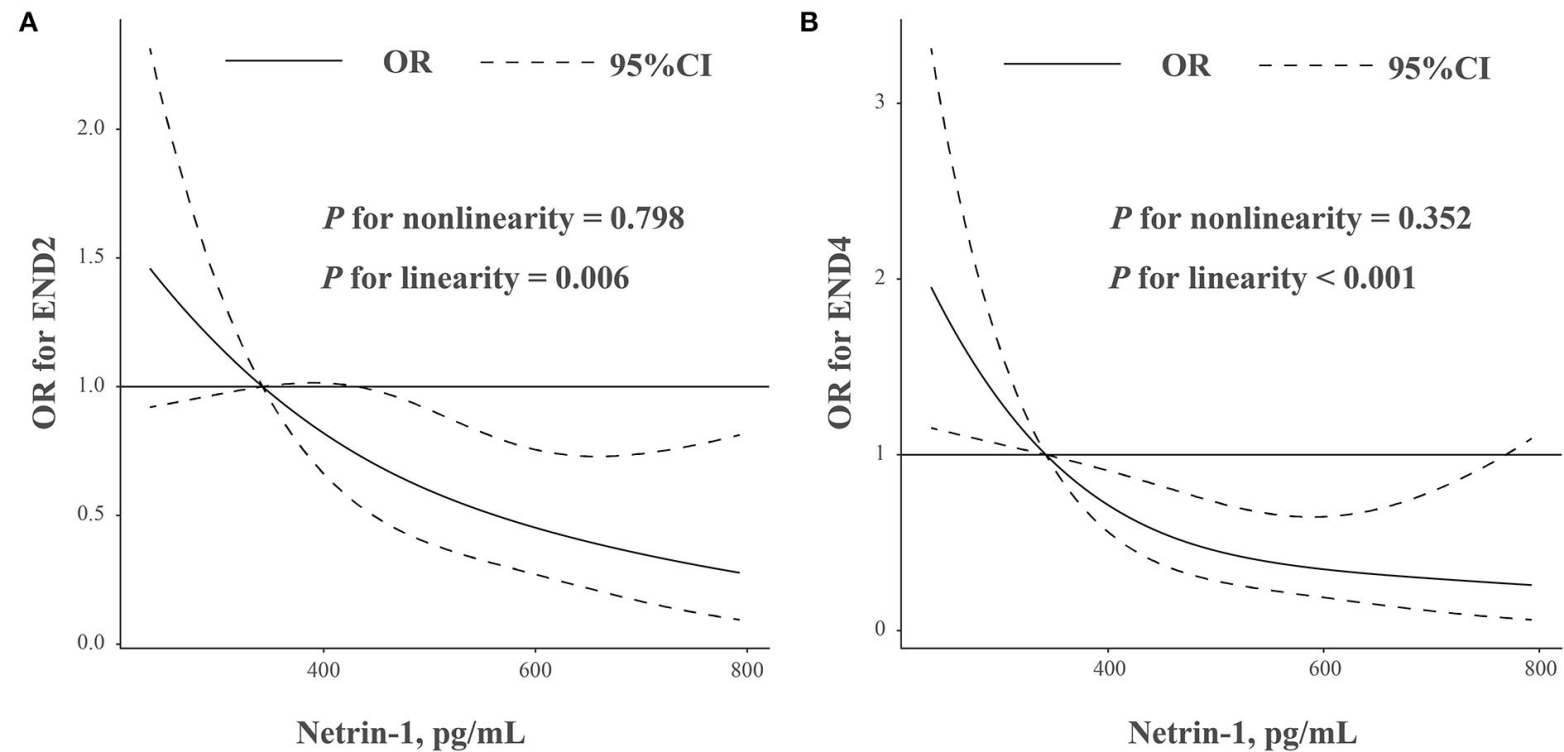
**(A,B)** Association was fitted with a restricted cubic spline with 3 knots (at 5th, 50th, and 95th percentiles) adjusted for demographic characteristics, hypertension, baseline NIHSS score, baseline glucose levels, and hyper-sensitive C-reactive protein levels. The reference point for netrin-1 levels is 315.5 pg/ml (lowest quantiles of netrin-1 levels). CI, confidence intervals; OR, odds ratio.

In addition, restricted cubic spline analysis showed a linear association between NT-1 concentrations and the risk of END (Figure 1A, END2, *p* = 0.006 for linearity; Figure 1B, END4, *p* < 0.001 for linearity).

## Discussion

Early neurological deterioration is a common complication of AIS. The major causes of END include hemorrhagic transformation, malignant edema, progressive stroke, and poststroke seizure (3, 23). The incidence of patients with END was vary widely in previous studies, depending on the definition used. In the present study, END was observed in 31.0% of patients, which is defined as a significant neurofunctional decline (increment of NIHSS score by two points) within a 72-h period after admission. This incidence of END was similar to the previous studies (2–4). The important finding of the present study was that decreased NT-1 levels were associated with a higher rate of END, independent of demographic characteristics, baseline NIHSS score, and other potential confounders.

Accumulating evidence indicated that NT-1 could be used to reflect risk and severity in cerebrovascular disease patients (15, 24–27). The previous cross-sectional studies found that NT-1 levels might represent a potential biomarker for reflecting the severity and prognosis of aneurysmal subarachnoid hemorrhage (24) and acute intracerebral hemorrhage (25). In the China Antihypertensive Trial of AIS, decreased baseline NT-1 levels were found to be associated with improved prognosis 3 months after ischemic stroke in a multivariate logistic regression model (15). These results are prominently supportive of the neurovascular protection role of NT-1 in ischemic injury. Herein, we performed a prospective study to evaluate the association between serum NT-1 and END. Our study recruited a homogeneous population of patients with stroke and used the two END definitions widely recommended by researchers. Also, comprehensive information about potential confounders was controlled in the multivariate regression analysis. All these advantages of this study confirmed the reliability of our findings. Our study further found that the NT-1 concentrations were closely correlated with the risk of END after ischemic stroke, and the association was dose-response.

Several pathophysiological mechanisms could explain the observed effect of NT-1 on neurological deterioration after ischemic stroke. First, NT-1 presents bifunctional effects on blood vessels through receptor-dependent pathways. In middle cerebral artery occlusion rats, intracerebroventricularly infusion of NT-1 could ameliorate the blood–brain barrier impairment secondary to ischemic stroke by promoting tight junction function and endothelial survival *via* activating the PI3K Pathway (13). Second, It is known that NT-1 is a laminin-related protein enriching axonal extension and regulating angiogenesis (8, 9). The growth of new capillary blood vessels increases the blood supply in the ischemic penumbra and functions as a scaffold to translate the neurons to the ischemic periphery (28). AAV-mediated netrin-1 overexpression also improves the peri-infarct vascular density, which might reduce the infarct size and improve functional recovery (14). In addition, systemic human NT-1 gene delivery by AAV can reduce leukocyte accumulation, which in turn inhibits neuroinflammation and brain parenchymal injury (29). Taken together, NT-1 might prevent neurological deterioration after ischemic stroke by mediating the permeability of the blood–brain barrier, endothelial function, inflammation, and angiogenesis. Further clinical trials are needed to assess whether patients with ischemic stroke could benefit from the exogenous NT-1 supplement.

Several limitations of this study merit consideration. First, due to the limitation inherent in the cross-sectional study, we could only show association, not causality. Second, NT-1 concentrations were measured only once after admission. NT-1 levels should be measured dynamically for longitudinal analysis, which might provide additional information on the development and its prognostic implications. Third, patients with AIS who received reperfusion therapy were excluded from this study, which might underestimate the actual incidence of END and limit the generalizability of our results. Finally, the previous studies used inconsistent definitions of END, leading to the discrepancy in incidence rates and associated factors. However, the 2 END definitions used in our study have been widely accepted and recommended by researchers.

In conclusion, AIS patients with decreased baseline NT-1 levels were easier to experience END during hospitalization, which may be an important indicator for risk stratification of END after AIS. Future studies are warranted to validate our findings and explore the detailed pathway of NT-1 in mediating the neurological fluctuation after ischemic stroke.

## Data availability statement

The original contributions presented in the study are included in the article/supplementary material, further inquiries can be directed to the corresponding author/s.

## Ethics statement

The studies involving human participants were reviewed and approved by the Ethics Review Board of Mianzhu People's Hospital. The patients/participants provided their written informed consent to participate in this study.

## Author contributions

JT and ZC designed and conceptualized the study, analyzed the data, interpreted the data, drafted, and revised the manuscript. TC, XZ, YW, WF, CF, YJ, QZ, JP, JL, ZY, and XY played a major role in the acquisition. All authors contributed to the article and approved the submitted version.

## Conflict of interest

The authors declare that the research was conducted in the absence of any commercial or financial relationships that could be construed as a potential conflict of interest.

## Publisher's note

All claims expressed in this article are solely those of the authors and do not necessarily represent those of their affiliated organizations, or those of the publisher, the editors and the reviewers. Any product that may be evaluated in this article, or claim that may be made by its manufacturer, is not guaranteed or endorsed by the publisher.
